# Hispanic ethnicity and mortality among critically ill patients with COVID-19

**DOI:** 10.1371/journal.pone.0268022

**Published:** 2022-05-18

**Authors:** Ana C. Ricardo, Jinsong Chen, Stephanie M. Toth-Manikowski, Natalie Meza, Min Joo, Shruti Gupta, Deepa G. Lazarous, David E. Leaf, James P. Lash, on behalf of the STOP-COVID Investigators

**Affiliations:** 1 Department of Medicine, Division of Nephrology, University of Illinois at Chicago, Chicago, IL, United States of America; 2 Department of Medicine, Division of Renal Medicine, Brigham and Women’s Hospital, Boston, MA, United States of America; 3 Department of Pulmonary Critical Care and Sleep Medicine, Georgetown University Hospital, Washington, DC, United States of America; The University of Tennessee Knoxville, UNITED STATES

## Abstract

**Background:**

Hispanic persons living in the United States (U.S.) are at higher risk of infection and death from coronavirus disease 2019 (COVID-19) compared with non-Hispanic persons. Whether this disparity exists among critically ill patients with COVID-19 is unknown.

**Objective:**

To evaluate ethnic disparities in mortality among critically ill adults with COVID-19 enrolled in the Study of the Treatment and Outcomes in Critically Ill Patients with COVID-19 (STOP-COVID).

**Methods:**

Multicenter cohort study of adults with laboratory-confirmed COVID-19 admitted to intensive care units (ICU) at 67 U.S. hospitals from March 4 to May 9, 2020. Multilevel logistic regression was used to evaluate 28-day mortality across racial/ethnic groups.

**Results:**

A total of 2153 patients were included (994 [46.2%] Hispanic and 1159 [53.8%] non-Hispanic White). The median (IQR) age was 62 (51–71) years (non-Hispanic White, 66 [57–74] years; Hispanic, 56 [46–67] years), and 1462 (67.9%) were men. Compared with non-Hispanic White patients, Hispanic patients were younger; were less likely to have hypertension, chronic obstructive pulmonary disease, coronary artery disease, or heart failure; and had longer duration of symptoms prior to ICU admission. During median (IQR) follow-up of 14 (7–24) days, 785 patients (36.5%) died. In analyses adjusted for age, sex, clinical characteristics, and hospital size, Hispanic patients had higher odds of death compared with non-Hispanic White patients (OR, 1.44; 95% CI, 1.12–1.84).

**Conclusions:**

Among critically ill adults with COVID-19, Hispanic patients were more likely to die than non-Hispanic White patients, even though they were younger and had lower comorbidity burden. This finding highlights the need to provide earlier access to care to Hispanic individuals with COVID-19, especially given our finding of longer duration of symptoms prior to ICU admission among Hispanic patients. In addition, there is a critical need to address ongoing disparities in post hospital discharge care for patients with COVID-19.

## Introduction

Hispanic persons living in the United States (U.S.) are at increased risk of coronavirus disease 2019 (COVID-19) infection. Although they constitute only 18.5% of the U.S. population, Hispanic individuals account for 26.2% of COVID-19 cases reported to the Centers for Disease Control and Prevention (CDC) as of October 24, 2021 [1]. However, there are conflicting data on whether COVID-19-related mortality is higher among Hispanic persons. Data from the CDC indicate a 2.3-fold higher risk of age-adjusted COVID-19-related death among Hispanic compared to non-Hispanic Whites [2], whereas a recent meta-analysis of data from observational studies predominantly conducted within a single health care system suggests that there is no disparity in COVID-19 case fatality by ethnicity [3]. However, these studies were not focused on critically ill patients. Therefore, we conducted a study to evaluate ethnic disparities in mortality among critically ill adults with COVID-19 enrolled in the Study of the Treatment and Outcomes in Critically Ill Patients with COVID-19 (STOP-COVID).

## Materials and methods

The design and methods of STOP-COVID have been previously published [4]. STOP-COVID enrolled 4422 consecutive adults (age ≥18 years) with laboratory-confirmed COVID-19 (detected by nasopharyngeal or oropharyngeal swab) admitted to intensive care units (ICU) at 67 U.S. hospitals (**S1 Table**) between March 4 and May 9, 2020. Of those, 994 (22.6%) were identified by manual review of electronic medical records as Hispanic; 1159 (26.3%) as non-Hispanic White; 1220 (27.7%) as non-Hispanic Black; 288 (6.5%) as Asian or another race; 543 (12.3%) had unknown/not reported race; and 203 (4.6%) had unknown/not reported ethnicity. For the current study, we included STOP-COVID participants who were identified as Hispanic (n = 996) or non-Hispanic White (n = 1159). Patients were followed until the first of hospital discharge, death, or June 5, 2020. The study was approved by the Institutional Review Board (IRB) of the STOP-COVID Coordinating Center (Mass General Brigham IRB), as well as the IRB of each of the 67 participating institutions with a waiver of informed consent.

Study personnel at each site collected data by manual review of electronic medical records using a standardized case report form [4] to enter data into REDCap (Research Electronic Data Capture), a secure online data collection tool [5]. Patient-level data included baseline demographics (sex [men or women], ethnicity [Hispanic or Latino, not Hispanic or Latino, or unknown/not reported], race [White, Black or African American, Asian, American Indian/Alaska Native, Native Hawaiian or Other Pacific Islander, more than one race, or unknown/not reported]), coexisting medical conditions (including diabetes mellitus, hypertension, chronic obstructive pulmonary disease [COPD], asthma, chronic kidney disease [CKD]), symptoms and medications prior to hospital admission, and vital signs on ICU admission. The definitions of baseline characteristics, comorbidities, treatments, and outcomes are presented in **S2 Table**. In addition, daily data for the 14 days following ICU admission were collected on physiologic and laboratory values, pharmacologic and non-pharmacologic treatments, and organ support.

The primary outcome was mortality within 28 days of ICU admission. Patients who were discharged from the hospital prior to 28 days were considered to be alive (we confirmed the validity of this assumption in a subset of patients, as described elsewhere) [4].

### Statistical analysis

Baseline characteristics for Hispanic and non-Hispanic White patients are summarized as mean (SD) or median (IQR) for continuous variables, and count (proportion) for categorical variables. Chi-squared test was used to compare categorical variables, and t-test or Wilcoxon rank sum test was used to compare continuous variables. To evaluate the association between ethnicity and mortality, we used mixed effects logistic regression with hospital as a random effect and other covariates as fixed effects. Regression models were adjusted for the following prespecified patient and hospital characteristics based on clinical knowledge and prior studies [4, 6–9]: age, sex, body mass index, smoking status, hypertension, diabetes, coronary artery disease, heart failure, COPD, CKD, duration of symptoms prior to ICU admission; d-dimer, ratio of PaO_2_ over the fraction of inspired oxygen, lymphocyte count, the renal component of the sequential organ failure assessment (SOFA) score on ICU admission; number of pre-COVID ICU beds; and medication use prior to hospital admission (angiotensin-converting enzyme inhibitor, angiotensin II receptor blocker, nonsteroidal anti-inflammatory drug, aspirin, and vitamin D) (**S2 Appendix**). Missing data were not imputed. Instead, we created a separate missing category for each covariate that had missing data (**Table 1**), since data may not have been missing at random [10, 11]. As a sensitivity analysis, we conducted Cox proportional hazards regression analysis to evaluate the association between ethnicity and mortality. All tests were 2-sided, and p<0.05 was considered statistically significant. Analyses were performed using SAS 9.4 (Cary, NC).

## Results

A total of 2153 patients were included (median [IQR] age, 62 [51–71] years; 1462 [67.9%] men; 994 [46.2%] Hispanic and 1159 [53.8%] non-Hispanic White). Baseline characteristics are shown in **Table 1**.

**Fig 1:**
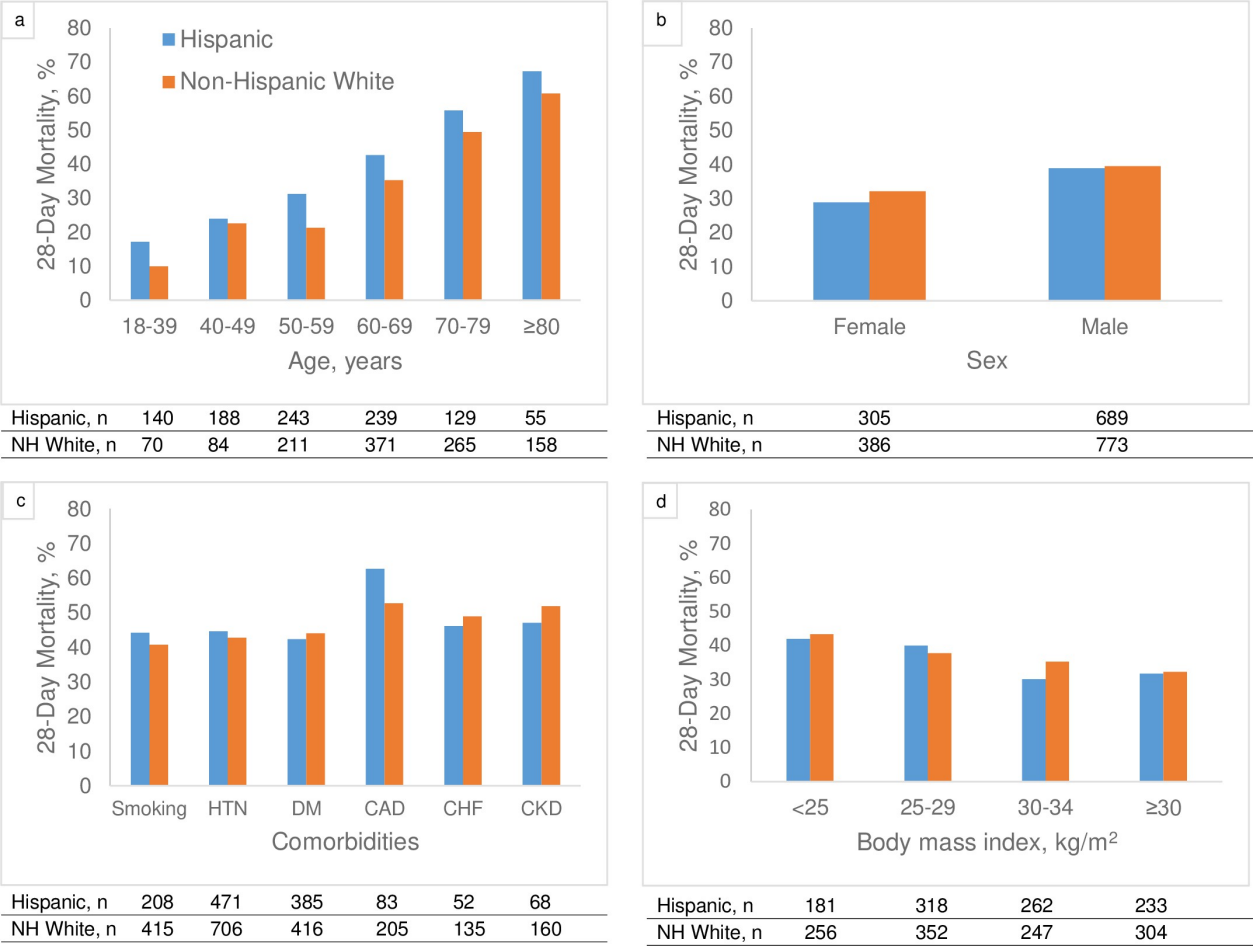
Unadjusted mortality rate by ethnicity, and pre-specified subgroups. Twenty-eight-day mortality by (a) age group, (b) sex, (c) comorbidities, and (d) body mass index. Abbreviations: CAD, coronary artery disease; CHF, congestive heart failure; CKD, chronic kidney disease; DM, diabetes; HTN, hypertension.

Compared with non-Hispanic White patients, Hispanic patients were younger (median [IQR] age 56 [46–67] vs. 66 [57–74] years), had lower prevalence of most major comorbid conditions, and were more likely to have markers of severe illness (**Table 1**). During a median follow-up of 14 days (IQR, 7–24), 785 patients (36.5%) died (356 [35.8%] Hispanic and 429 [37.0%] non-Hispanic White). Twenty-eight day mortality rate was higher among Hispanic patients compared with non-Hispanic White patients for each age strata (**Fig 1**). Mortality rates by sex, comorbid conditions, and body mass index are shown in **Fig 1**. In fully-adjusted models, Hispanic patients had higher odds of death compared with non-Hispanic White patients (OR, 1.44; 95% CI, 1.12–1.84). The corresponding OR and 95% CI for each covariate included in the logistic regression model for death at 28 days are shown in **S3 Table**. Cox proportional regression analyses yielded similar results (multivariable-adjusted HR 1.27, 95% CI 1.08–1.51).

**Table 1:** Characteristics of adults with coronavirus disease 2019 admitted to the intensive care unit.

Characteristic	Patients, No. (%), mean (SD) or median (IQR)	
	Non-Hispanic White (N = 1159)	Hispanic (n = 994)
**Demographics**		
Age, years	66 (57–74)	56 (46–67)^a^
Sex		
Women	386 (33.3)	305 (30.7)
Men	773 (66.7)	689 (69.3)
Current or former smoker	415 (35.8)	208 (21.0)^a^
Body mass index categories, kg/m^2^		
<30	563 (48.6)	460 (46.3)
≥30	551 (47.5)	495 (49.8)
Missing	45 (3.9)	39 (3.9)
Symptom duration prior to ICU admission	7.73 (5.8)	8.58 (6.2)^a^
**Comorbidities**		
Diabetes	416 (35.9)	385 (38.7)
Hypertension	706 (60.9)	471 (47.4)^a^
Chronic obstructive pulmonary disease	147 (12.7)	25 (2.5)^a^
Coronary artery disease	205 (17.7)	83 (8.4)^a^
Congestive heart failure	135 (11.7)	52 (5.2)^a^
Chronic kidney disease	160 (13.8)	68 (6.8)^a^
**Vital signs on the day of ICU admission**		
Highest temperature, °C	37.9 (37.2,38.8)	38.1 (37.2,38.9)^a^
Lowest systolic blood pressure, mm Hg	97 (85–109)	96 (86–110)^a^
Highest heart rate, /min	102 (88–118)	107 (92–122)^a^
**Laboratory findings on the day of ICU admission** ^**b**^		
Lymphocyte count, /μL		
< 1000	682 (58.8)	509 (51.2)^a^
≥ 1000	249 (21.5)	323 (32.5)^a^
Missing	228 (19.7)	162 (16.3)
Serum Creatinine, mg/dL	1.06 (0.80,1.51)	0.90 (0.70,1.31)^a^
D-dimer, ng/mL		
<1000	256 (22.1)	270 (27.2)^a^
1000–2500	170 (14.7)	158 (15.9)^a^
>2500	170 (14.7)	181 (18.2)^a^
Missing	563 (48.6)	385 (38.7)
**Severity of illness on the day of ICU admission**		
Vasopressor use	446 (36.6)	432 (43.5)
Non-invasive mechanical ventilation	25 (2.2)	15 (1.5)
High-flow nasal cannula or nonrebreather mask	289 (25.0)	201 (20.4)^a^
Invasive mechanical ventilation	700 (60.7)	655 (66.4)^a^
FiO_2_	80 (58–100)	90 (60–100)^a^
PEEP, cm H_2_O	12 (10–15)	14 (10–16)^a^
PaO_2_:FiO_2_, mm Hg		
Not mechanically ventilated	454 (39.2)	331 (33.3)^a^
>200	159 (13.7)	120 (12.1)^a^
100–199	239 (20.6)	240 (24.1)^a^
<100	183 (15.8)	203 (20.4)^a^
Missing	124 (10.7)	100 (10.1)
**Antiviral and anti-inflammatory medications** ^**c**^		
Remdesivir	75 (6.5)	84 (8.5)
Any corticosteroid	421 (36.3)	398 (40.4)
Tocilizumab	196 (16.9)	194 (19.5)
**Clinical trial enrollment** ^**d**^	204 (17.7)	210 (21.3)^a^
**Hospital size (no. pre-COVID ICU beds)**		
Small (<50)	388 (33.5)	358 (36.0)
Medium (50–99)	309 (26.7)	299 (30.1)
Large (≥100)	462 (39.9)	337 (33.9)^a^

^a^P<0.05.

^b^Data regarding serum creatinine was missing for 98 (4.6%) patients.

^c^Received at any time within 14 days following ICU admission.

^d^Enrollment in a pharmacologic or nonpharmacologic clinical trial for COVID-19.

Abbreviations: ICU, intensive care unit; IQR, interquartile range; PaO_2_:FIO_2_, ratio of PaO_2_ over the fraction of inspired oxygen (assessed only in patients receiving invasive mechanical ventilation); PEEP, positive end-expiratory pressure.

SI conversion factor: to convert creatinine to micromoles per liter, multiply by 88.4.

## Discussion

In this large, multicenter U.S. cohort study of critically ill patients with COVID-19, we found that Hispanic patients were at higher risk of death despite being, on average, 10 years younger, and having a lower comorbidity burden compared with non-Hispanic White patients. These findings remained significant despite extensive adjustment for clinical characteristics.

Based on registry data, the COVID-19 mortality rate among Hispanic persons in higher than in non-Hispanic White persons [2, 3]. In contrast, clinical studies have shown no significant difference in mortality between Hispanic and non-Hispanic White patients who tested positive for COVID-19 [12, 13], or among those who were hospitalized with COVID-19 [3, 14–16]. In our study, we observed that among critically ill patients with COVID-19, Hispanic patients were at higher risk of death than non-Hispanic White patients. Reasons for our findings are unclear, but are likely complex and multifactorial. In the general U.S. population, the risk of mortality among Hispanic persons is lower than in non-Hispanic White persons [17], despite the limited access to quality health care, and the high comorbidity burden among Hispanic adults. This phenomenon is often referred to as the “Hispanic paradox”, and has been attributed to strong social support and other favorable social and behavioral factors [18, 19]. The Hispanic survival advantage was not observed in our study of critically ill adults with COVID-19. Potential underlying causes of this striking health disparity include social determinants of health, racism and discrimination, economic disadvantages and, access to quality health care [20–22]. This constellation of adverse factors are known to influence COVID-19 exposure and severity of clinical course. For example, living in crowded conditions and holding jobs that require the use of public transportation, as is often the case among Hispanic adults, is associated with exposure to higher levels of SARS-CoV-2. This is important because higher viral loads have been associated with disease severity and increased mortality [23]. Furthermore, although all patients in our study had access to intensive care, the duration of symptoms prior to ICU admission was significantly longer among Hispanic compared with non-Hispanic White patients (8.6 vs. 7.7 days), suggesting a delay in severe disease recognition, limited access to health care, or other unmeasured factors. In our analysis, the higher mortality risk among Hispanic patients persisted despite adjusting for this and other important factors, but whether other disparities in the timing and quality of care played a role remains unclear. Of note, we did not observe significant differences in the use of remdesivir, corticosteroids and tocilizumab between Hispanic and non-Hispanic White patients. However, our study was conducted early on during the pandemic, before therapies for COVID-19 were widely studied and/or available. Therefore, we were not able to fully evaluate the impact of inequitable use of effective medications, such as monoclonal antibodies [24], on ethnicity-related differences in mortality.

The results of our study need to be interpreted in light of its limitations. Although the U.S. Hispanic population is heterogeneous, our study was not able to evaluate whether risk for mortality varies by specific Hispanic background groups (e.g. Mexican, Puerto Rican, Dominican). In addition, patients were identified as Hispanic based on manual review of electronic medical records, instead of self-report, which could have led to misclassification of the exposure. Moreover, we did not have information regarding important social determinants of health, acculturation, or primary language.

In summary, among critically ill adults with COVID-19, Hispanics were at higher mortality risk than non-Hispanic Whites, even though they were younger and had a lower comorbidity burden. These findings highlight the need to urgently address the ongoing racial/ethnic disparities in equitable use of effective medications, as well as COVID-19 vaccination distribution by focusing efforts on Hispanic communities with high rates of COVID-19 infection and mortality. Moreover, Hispanic individuals with COVID-19 in these communities should be provided with better and more timely access to health care, including adequate access to post-discharge clinics, low cost or free medications for those without health insurance coverage, and discharge instructions translated to Spanish as needed.

## Supporting information

S1 Appendix STOP-COVID investigators. (DOCX)

S2 Appendix Supplemental methods. (DOCX)

S1 Table List of participating sites. (DOCX)

S2 Table Definitions of baseline characteristics, comorbidities, treatments, and outcomes. (DOCX)

S3 Table Multivariable-adjusted risk model for death at 28 days. (DOCX)

## References

[1] Centers for Disease Control and Prevention. Demographic Trends of COVID-19 cases and deaths in the US reported to CDC. Available: https://covid.cdc.gov/covid-data-tracker/#demographics.

[2] Centers for Disease Control and Prevention. Risk for COVID-19 Infection, Hospitalization, and Death By Race/Ethnicity. 11 Sep 2021 [cited 25 Oct 2021]. Available: https://www.cdc.gov/coronavirus/2019-ncov/covid-data/investigations-discovery/hospitalization-death-by-race-ethnicity.html.

[3] Mackey K, Ayers CK, Kondo KK, Saha S, Advani SM, Young S, et al. Racial and Ethnic Disparities in COVID-19-Related Infections, Hospitalizations, and Deaths: A Systematic Review. Ann Intern Med. 2021;174: 362–373. doi: 10.7326/M20-6306 33253040 PMC7772883

[4] Gupta S, Hayek SS, Wang W, Chan L, Mathews KS, Melamed ML, et al. Factors Associated With Death in Critically Ill Patients With Coronavirus Disease 2019 in the US. JAMA Intern Med. 2020;180: 1436–1447. doi: 10.1001/jamainternmed.2020.3596 32667668 PMC7364338

[5] Harris PA, Taylor R, Thielke R, Payne J, Gonzalez N, Conde JG. Research electronic data capture (REDCap)—a metadata-driven methodology and workflow process for providing translational research informatics support. J Biomed Inform. 2009;42: 377–381. doi: 10.1016/j.jbi.2008.08.010 18929686 PMC2700030

[6] Docherty AB, Harrison EM, Green CA, Hardwick HE, Pius R, Norman L, et al. Features of 20 133 UK patients in hospital with covid-19 using the ISARIC WHO Clinical Characterisation Protocol: prospective observational cohort study. BMJ. 2020;369: m1985. doi: 10.1136/bmj.m1985 32444460 PMC7243036

[7] Williamson EJ, Walker AJ, Bhaskaran K, Bacon S, Bates C, Morton CE, et al. OpenSAFELY: factors associated with COVID-19 death in 17 million patients. Nature. 2020. doi: 10.1038/s41586-020-2521-4 32640463 PMC7611074

[8] Price-Haywood EG, Burton J, Fort D, Seoane L. Hospitalization and Mortality among Black Patients and White Patients with Covid-19. New England Journal of Medicine. 2020;382: 2534–2543. doi: 10.1056/NEJMsa2011686 32459916 PMC7269015

[9] Albitar O, Ballouze R, Ooi JP, Ghadzi SMS. Risk factors for mortality among COVID-19 patients. Diabetes Research and Clinical Practice. 2020;166. doi: 10.1016/j.diabres.2020.108293 32623035 PMC7332436

[10] Gupta S, Coca SG, Chan L, Melamed ML, Brenner SK, Hayek SS, et al. AKI Treated with Renal Replacement Therapy in Critically Ill Patients with COVID-19. J Am Soc Nephrol. 2020. doi: 10.1681/ASN.2020060897 33067383 PMC7894677

[11] Gupta S, Wang W, Hayek SS, Chan L, Mathews KS, Melamed ML, et al. Association Between Early Treatment With Tocilizumab and Mortality Among Critically Ill Patients With COVID-19. JAMA Intern Med. 2020. doi: 10.1001/jamainternmed.2020.6252 33080002 PMC7577201

[12] Rentsch CT, Kidwai-Khan F, Tate JP, Park LS, King JT, Skanderson M, et al. Patterns of COVID-19 testing and mortality by race and ethnicity among United States veterans: A nationwide cohort study. PLoS Med. 2020;17: e1003379. doi: 10.1371/journal.pmed.1003379 32960880 PMC7508372

[13] Pan AP, Khan O, Meeks JR, Boom ML, Masud FN, Andrieni JD, et al. Disparities in COVID-19 hospitalizations and mortality among black and Hispanic patients: cross-sectional analysis from the greater Houston metropolitan area. BMC Public Health. 2021;21: 1330. doi: 10.1186/s12889-021-11431-2 34229621 PMC8258471

[14] Golestaneh L, Neugarten J, Fisher M, Billett HH, Gil MR, Johns T, et al. The association of race and COVID-19 mortality. EClinicalMedicine. 2020;25: 100455. doi: 10.1016/j.eclinm.2020.100455 32838233 PMC7361093

[15] Kim L, Garg S, O’Halloran A, Whitaker M, Pham H, Anderson EJ, et al. Risk Factors for Intensive Care Unit Admission and In-hospital Mortality Among Hospitalized Adults Identified through the US Coronavirus Disease 2019 (COVID-19)-Associated Hospitalization Surveillance Network (COVID-NET). Clin Infect Dis. 2021;72: e206–e214. doi: 10.1093/cid/ciaa1012 32674114 PMC7454425

[16] Petrilli CM, Jones SA, Yang J, Rajagopalan H, O’Donnell L, Chernyak Y, et al. Factors associated with hospital admission and critical illness among 5279 people with coronavirus disease 2019 in New York City: prospective cohort study. BMJ. 2020;369: m1966. doi: 10.1136/bmj.m1966 32444366 PMC7243801

[17] Ruiz JM, Steffen P, Smith TB. Hispanic mortality paradox: a systematic review and meta-analysis of the longitudinal literature. Am J Public Health. 2013;103: e52–60. doi: 10.2105/AJPH.2012.301103 23327278 PMC3673509

[18] Abraído-Lanza AF, Mendoza-Grey S, Flórez KR. A Commentary on the Latin American Paradox. JAMA Netw Open. 2020;3: e1921165. doi: 10.1001/jamanetworkopen.2019.21165 32049288

[19] Abraído-Lanza AF, Dohrenwend BP, Ng-Mak DS, Turner JB. The Latino mortality paradox: a test of the “salmon bias” and healthy migrant hypotheses. Am J Public Health. 1999;89: 1543–1548. doi: 10.2105/ajph.89.10.1543 10511837 PMC1508801

[20] Webb Hooper M, Nápoles AM, Pérez-Stable EJ. COVID-19 and Racial/Ethnic Disparities. JAMA. 2020;323: 2466–2467. doi: 10.1001/jama.2020.8598 32391864 PMC9310097

[21] Lopez L, Hart LH, Katz MH. Racial and Ethnic Health Disparities Related to COVID-19. JAMA. 2021;325: 719–720. doi: 10.1001/jama.2020.26443 33480972

[22] Macias Gil R, Marcelin JR, Zuniga-Blanco B, Marquez C, Mathew T, Piggott DA. COVID-19 Pandemic: Disparate Health Impact on the Hispanic/Latinx Population in the United States. J Infect Dis. 2020;222: 1592–1595. doi: 10.1093/infdis/jiaa474 32729903 PMC7454709

[23] Fajnzylber J, Regan J, Coxen K, Corry H, Wong C, Rosenthal A, et al. SARS-CoV-2 viral load is associated with increased disease severity and mortality. Nat Commun. 2020;11: 5493. doi: 10.1038/s41467-020-19057-5 33127906 PMC7603483

[24] Wiltz JL. Racial and Ethnic Disparities in Receipt of Medications for Treatment of COVID-19—United States, March 2020–August 2021. MMWR Morb Mortal Wkly Rep. 2022;71. doi: 10.15585/mmwr.mm7103e1 35051133 PMC8774154

