# Supplementary material for: Hispanic ethnicity and mortality among critically ill patients with COVID-19
Source: PLoS One. 2022 May 18;17(5):e0268022. doi: 10.1371/journal.pone.0268022 (PMC9116663; doi:10.1371/journal.pone.0268022)
Supplement: S1 Appendix — (DOCX) [file pone.0268022.s001.docx]

**Supplemental Information**

**S1 Appendix. STOP-COVID Investigators**

**Baylor College of Medicine:** Carl P. Walther*, Samaya J. Anumudu

**Baylor University Medical Center:** Justin Arunthamakun*, Kathleen F. Kopecky, Gregory P. Milligan, Peter A. McCullough, Thuy-Duyen Nguyen

**Beth Israel Deaconess Medical Center:** Shahzad Shaefi*, Megan L. Krajewski, Sidharth Shankar, Ameeka Pannu, Juan D. Valencia

**Boston Medical Center:** Sushrut S. Waikar*, Zoe A. Kibbelaar

**Cook County Health:** Ambarish M. Athavale*, Peter Hart, Shristi Upadhyay, Ishaan Vohra, Ajiboye Oyintayo

**Cooper University Health Care:** Adam Green*, Jean-Sebastien Rachoin, Christa A. Schorr, Lisa Shea

**Duke University Medical Center:** Daniel L. Edmonston*, Christopher L. Mosher

**Hackensack Meridian Health Mountainside Medical Center:** Alexandre M. Shehata*, Zaza Cohen, Valerie Allusson, Gabriela Bambrick-Santoyo, Noor ul aain Bhatti, Bijal Mehta, Aquino Williams

**Hackensack Meridian Health Hackensack University Medical Center:** Samantha K. Brenner*, Patricia Walters, Ronaldo C. Go, Keith M. Rose

**Harvard T.H. Chan School of Public Health:** Miguel A. Hernán

**Harvard University:** Amy M. Zhou, Ethan C. Kim, Rebecca Lisk

**Icahn School of Medicine at Mount Sinai:** Lili Chan*, Kusum S. Mathews*, Steven G. Coca, Deena R. Altman, Aparna Saha, Howard Soh, Huei Hsun Wen, Sonali Bose, Emily A. Leven, Jing G. Wang, Gohar Mosoyan, Girish N. Nadkarni, Pattharawin Pattharanitima, Emily J. Gallagher

**Indiana University School of Medicine/Indiana University Health:** Allon N. Friedman*, John Guirguis, Rajat Kapoor, Christopher Meshberger, Katherine J. Kelly

**Johns Hopkins Hospital:** Chirag R. Parikh*, Brian T. Garibaldi, Celia P. Corona-Villalobos, Yumeng Wen, Steven Menez, Rubab F. Malik, Carmen Elena Cervantes, Samir C. Gautam

**Kings County Hospital Center**: Mary C. Mallappallil*, Jie Ouyang, Sabu John, Ernie Yap, Yohannes Melaku, Ibrahim Mohamed, Siddhartha Bajracharya, Isha Puri, Mariah Thaxton, Jyotsna Bhattacharya, John Wagner, Leon Boudourakis

**Loma Linda University:** H. Bryant Nguyen*, Afshin Ahoubim

**Mayo Clinic, Arizona:** Leslie F. Thomas*, Dheeraj Reddy Sirganagari

**Mayo Clinic, Florida:** Pramod K. Guru*

**Mayo Clinic, Rochester:** Kianoush Kashani*, Shahrzad Tehranian

**Medical College of Wisconsin:** Yan Zhou,* Paul A. Bergl, Jesus Rodriguez, Jatan A. Shah, Mrigank S. Gupta

**MedStar Georgetown University Hospital:** Princy N. Kumar*, Deepa G. Lazarous, Seble G. Kassaye

**Montefiore Medical Center/Albert Einstein College of Medicine**: Michal L. Melamed*, Tanya S. Johns, Ryan Mocerino, Kalyan Prudhvi, Denzel Zhu, Rebecca V. Levy, Yorg Azzi, Molly Fisher, Milagros Yunes, Kaltrina Sedaliu, Ladan Golestaneh, Maureen Brogan, Neelja Kumar, Michael Chang, Jyotsana Thakkar

**New York-Presbyterian Queens Hospital**: Ritesh Raichoudhury*, Akshay Athreya, Mohamed Farag

**New York-Presbyterian/Weill Cornell Medical Center:** Edward J. Schenck*, Soo Jung Cho, Maria Plataki, Sergio L. Alvarez-Mulett, Luis G. Gomez-Escobar, Di Pan, Stefi Lee, Jamuna Krishnan, William Whalen

**New York University Langone Hospital:** David Charytan*, Ashley Macina, Sobaata Chaudhry, Benjamin Wu, Frank Modersitzki

**Northwestern Memorial Hospital:** Northwestern University Feinberg School of Medicine - Anand Srivastava*, Alexander S. Leidner, Carlos Martinez, Jacqueline M. Kruser, Richard G. Wunderink, Alexander J. Hodakowski

**Ochsner Medical Center:** Juan Carlos Q. Velez*, Eboni G. Price-Haywood, Luis A. Matute-Trochez, Anna E. Hasty, Muner MB. Mohamed

**Oregon Health and Science University Hospital:** Rupali S. Avasare*, David Zonies*

**Partners Healthcare:** Brigham and Women’s Hospital, Brigham and Women’s Faulkner Hospital, Massachusetts General Hospital, and Newton Wellesley Hospital - David E. Leaf*, Shruti Gupta*, Meghan E. Sise, Erik T. Newman, Samah Abu Omar, Kapil K. Pokharel, Shreyak Sharma, Harkarandeep Singh, Simon Correa, Tanveer Shaukat, Omer Kamal, Wei Wang, Heather Yang, Jeffery O. Boateng, Meghan Lee, Ian A. Strohbehn, Jiahua Li, Ariel L. Mueller

**ProMedica Health System**: Roberta E. Redfern,* Nicholas S. Cairl, Gabriel Naimy, Abeer Abu-Saif, Danyell Hall, Laura Bickley

**Renown Health:** Chris Rowan*, Farah Madhani-Lovely*, Vivian S. Cruz, Kristen M. Hess, Alanna L. Jacobs

**Rush University Medical Center:** Vasil Peev*, Jochen Reiser, John J. Byun, Andrew Vissing, Esha M. Kapania, Zoe Post, Nilam P. Patel, Joy-Marie Hermes

**Rutgers/New Jersey Medical School:** Anne K. Sutherland*, Amee Patrawalla, Diana G. Finkel, Barbara A. Danek, Sowminya Arikapudi, Jeffrey M. Paer, Peter Cangialosi, Mark Liotta

**Rutgers/Robert Wood Johnson Medical School:** Jared Radbel*, Sonika Puri, Jag Sunderram, Matthew T. Scharf, Ayesha Ahmed, Ilya Berim, Jayanth S. Vatson

**Stanford Healthcare:** Stanford University School of Medicine – Shuchi Anand*, Joseph E. Levitt, Pablo Garcia

**Temple University Hospital:** Suzanne M. Boyle*, Rui Song

**Thomas Jefferson University Hospital**: Jingjing Zhang*, Sang Hoon Woo, Xiaoying Deng, Goni Katz-Greenberg, Katharine Senter

**Tulane Medical Center:** Moh’d A. Sharshir*, Vadym V. Rusnak

**United Health Services Hospitals:** Muhammad Imran Ali

**University of Colorado Anschutz Medical Campus:** Anip Bansal*, Amber S. Podoll, Michel Chonchol, Sunita Sharma, Ellen L. Burnham, David J. Douin

**University Hospitals Cleveland Medical Center:** Arash Rashidi*, Rana Hejal

**University of Alabama-Birmingham Hospital:** Eric Judd*, Laura Latta, Ashita Tolwani

**University of California-Davis Medical Center:** Timothy E. Albertson*, Jason Y. Adams

**University of California-Los Angeles Medical Center:** Ronald Reagan-UCLA Medical Center - Steven Y. Chang*, Rebecca M. Beutler; UCLA Medical Center, Santa Monica – Carl E. Schulze

**University of California-San Diego Medical Center:** Etienne Macedo*, Harin Rhee

**University of California-San Francisco Medical Center:** Kathleen D. Liu*, Vasantha K. Jotwani

**University of Chicago Medical Center:** Jay L. Koyner*

**University of Florida Health-Gainesville:** Chintan V. Shah*

**University of Florida-Health-Jacksonville:** Vishal Jaikaransingh*

**University of Illinois Hospital and Health Sciences System:** Stephanie M. Toth-Manikowski*, Min J. Joo*, James P. Lash

**University of Kentucky Medical Center:** Javier A. Neyra*, Nourhan Chaaban, Madona Elias, Yahya Ahmad

**University Medical Center of Southern Nevada:** Rajany Dy*, Alfredo Iardino, Elizabeth H. Au, Jill H. Sharma

**University of Miami Health System:** Marie Anne Sosa*, Sabrina Taldone, Gabriel Contreras, David De La Zerda, Hayley B. Gershengorn, Bhavarth Shukla, Alessia Fornoni, Tanira Ferreira

**University of Michigan:** Salim S. Hayek*, Pennelope Blakely, Hanna Berlin, Tariq U. Azam, Husam Shadid, Michael Pan, Patrick O’ Hayer, Chelsea Meloche, Rafey Feroze, Rayan Kaakati, Danny Perry, Abbas Bitar, Elizabeth Anderson, Kishan J. Padalia, John P. Donnelly, Andrew J. Admon

**University of North Carolina School of Medicine:** Jennifer E. Flythe*, Matthew J. Tugman, Emily H. Chang

**University of Oklahoma Health Sciences Center:** Brent R. Brown*

**University of Pennsylvania Health System:** Amanda K. Leonberg-Yoo*, Ryan C. Spiardi, Todd A. Miano, Meaghan S. Roche, Charles R. Vasquez

**University of Pittsburgh Medical Center:** Amar D. Bansal*, Natalie C. Ernecoff, Sanjana Kapoor, Siddharth Verma, Huiwen Chen

**University of Tennessee Health Science Center and Memphis VA Medical Center/Methodist**

**University Hospital** – Csaba P. Kovesdy*, Miklos Z. Molnar*, Ambreen Azhar

**University of Texas Southwestern Medical Center and Parkland Health and Hospital System:** S. Susan Hedayati*, Mridula V. Nadamuni, Shani Shastri, Duwayne L. Willett

**University of Vermont Larner College of Medicine:** Samuel A.P. Short

**University of Virginia Health System:** Amanda D. Renaghan*, Kyle B. Enfield

**University of Washington Medical Center:** Pavan K. Bhatraju*, A. Bilal Malik

**Vanderbilt University Medical Center:** Matthew W. Semler

**Washington University in St. Louis/Barnes Jewish Hospital**: Anitha Vijayan*, Christina Mariyam Joy, Tingting Li, Seth Goldberg, Patricia F. Kao

**Wellforce Health System:** Lowell General Hospital **-** Greg L. Schumaker*, Tufts Medical Center - Nitender Goyal*, Anthony J. Faugno, Greg L. Schumaker, Caroline M. Hsu, Asma Tariq, Leah Meyer, Ravi K. Kshirsagar, Daniel E. Weiner, Aju Jose

**Westchester Medical Center:** Marta Christov*, Jennifer Griffiths, Sanjeev Gupta, Aromma Kapoor, Savneek Chugh

**Yale School of Medicine:** Perry Wilson,* Tanima Arora, Ugochukwu Ugwuowo

*Site Principal Investigator
