# Supplementary material for: Hispanic ethnicity and mortality among critically ill patients with COVID-19
Source: PLoS One. 2022 May 18;17(5):e0268022. doi: 10.1371/journal.pone.0268022 (PMC9116663; doi:10.1371/journal.pone.0268022)
Supplement: S2 Appendix — (DOCX) [file pone.0268022.s002.docx]

**S2 Appendix. Supplemental Methods**

**Multivariable modeling of the association between Hispanic ethnicity and mortality**

We performed multivariable regression modeling to estimate the association between Hispanic ethnicity (vs. non-Hispanic White) and 28-day mortality. We prespecified the following variables for inclusion in the multivariable model based on clinical knowledge and completeness of data:

1. Age (continuous)
2. Male sex
3. Body mass index (<30 vs. ≥30 kg/m^2^)
4. Smoking status (current/former vs. never)
5. Chronic kidney disease
6. Hypertension
7. Diabetes
8. Coronary artery disease
9. Heart failure
10. Chronic obstructive pulmonary disease
11. Duration of symptoms prior to ICU admission in days (continuous)
12. D-dimer in ng/mL (<1000 vs 1000-2500 or >2500, missing*)
13. Ratio of PaO_2_ over the fraction of inspired oxygen on ICU day 1 (Not ventilated vs ≥200, 100-199 or <100, missing*)
14. Lymphocyte per µL (< 1000 vs ≥ 1000, missing*)
15. Number of pre-COVID ICU beds (50-99 vs <50 or ≥100)
16. Medication use prior to hospital admission (angiotensin-converting enzyme inhibitor, angiotensin II receptor blocker, nonsteroidal anti-inflammatory drug, aspirin, and vitamin D)
17. Renal component of the Sequential Organ Failure Assessment (SOFA) score as follows:

|  |  | **Categories** | | | |
| --- | --- | --- | --- | --- | --- |
|  | **0** | **1** | **2** | **3** | **4** |
| SOFA Renal (Cr, UOP, and acute RRT) | Cr<1.2 and UOP≥500 | Cr 1.2-1.9 and UOP≥500 | Cr 2-3.4 and UOP≥500 | Cr 3.5-4.9 or UOP<500 | Cr ≥5 or UOP<200 or acute RRT or ESRD |

*Abbreviations: Cr, creatinine; ESRD, end stage renal disease; RRT, renal replacement therapy; UOP, urine output*

*Missing data were not imputed. Instead, we created a separate missing category for each covariate that had missing data (shown in Table 1 of the manuscript), since data may not have been missing at random.
