# Supplementary material for: Hispanic ethnicity and mortality among critically ill patients with COVID-19
Source: PLoS One. 2022 May 18;17(5):e0268022. doi: 10.1371/journal.pone.0268022 (PMC9116663; doi:10.1371/journal.pone.0268022)
Supplement: S1 Table — (DOCX) [file pone.0268022.s003.docx]

**S1 Table. List of Participating Sites**

| **Northeast** |
| --- |
| Beth Israel Deaconess Medical Center |
| Brigham and Women’s Faulkner Hospital |
| Brigham and Women's Hospital |
| Cooper University Health Care |
| Hackensack Meridian Health Hackensack University Medical Center |
| Hackensack Mountainside Hospital |
| Johns Hopkins Hospital |
| Kings County Hospital Center |
| Lowell General Hospital |
| Massachusetts General Hospital |
| MedStar Georgetown University Hospital |
| Montefiore Medical Center |
| Mount Sinai |
| Newton Wellesley Hospital |
| New York-Presbyterian Queens Hospital |
| New York-Presbyterian/Weill Cornell Medical Center |
| New York University Langone Hospital |
| Rutgers/New Jersey Medical School |
| Rutgers/Robert Wood Johnson Medical School |
| Temple University Hospital |
| Thomas Jefferson University Hospital |
| Tufts Medical Center |
| United Health Services Hospitals |
| University of Pennsylvania Health System |
| University of Pittsburgh Medical Center |
| Westchester Medical Center |
| Yale University Medical Center |
| **South** |
| Baylor College of Medicine, Houston |
| Baylor University Medical Center/Baylor Scott White and Health |
| Duke University Medical Center |
| Mayo Clinic, Florida |
| Memphis VA Medical Center |
| Methodist University Hospital |
| Ochsner Medical Center |
| Tulane Medical Center |
| University of Alabama-Birmingham Hospital |
| University of Florida Health-Gainesville |
| University of Florida Health-Jacksonville |
| University of Miami Health System |
| University of North Carolina Hospitals |
| University of Texas Southwestern Medical Center |
| University of Virginia Health System |
| **Midwest** |
| Barnes-Jewish Hospital |
| Cook County Health |
| Froedtert Hospital |
| Indiana University Health Methodist Hospital |
| Northwestern Memorial Hospital |
| Promedica Health System |
| Rush University Medical Center |
| University Hospitals Cleveland Medical Center |
| University of Chicago Medical Center |
| University of Illinois Hospital and Health Sciences System |
| University of Kentucky Hospital |
| University of Michigan Hospital |
| University of Oklahoma Health Sciences Center |
| **West** |
| Loma Linda University Medical Center |
| Mayo Clinic, Arizona |
| Oregon Health and Science University Hospital |
| Renown Health |
| Stanford Healthcare |
| University of California-Davis Medical Center |
| University of California-Los Angeles Medical Center |
| University of California-San Diego Medical Center |
| University of California-San Francisco Medical Center |
| UCHealth University of Colorado |
| University Medical Center of Southern Nevada |
| University of Washington Medical Center |

**S2 Table. Definitions of Baseline Characteristics, Comorbidities, Treatments, and Outcomes**

| **Baseline Characteristics** |  |
| --- | --- |
| Baseline serum creatinine | Lowest value (mg/dl) within 365 to 7 days prior to hospital admission. If not available, serum creatinine on hospital admission |
| Home medications | Medications that the patient was taking at home within 1 week prior to admission. Does not include those started at an outside hospital if the patient was transferred. |
| **Coexisting Conditions** |  |
| Asthma | Per chart review |
| Chronic kidney disease | Baseline eGFR< 60 on at least two consecutive values at least 12 weeks apart prior  to hospital admission. If not available, defined as per chart review. |
| Chronic liver disease | Cirrhosis, alcohol-related liver disease, nonalcoholic fatty liver disease, autoimmune hepatitis, hepatitis B or hepatitis C, primary biliary cirrhosis, or other |
| Chronic obstructive pulmonary disease | Per chart review |
| Congestive heart failure | Per chart review; heart failure with preserved versus reduced ejection fraction |
| Coronary artery disease | Per chart review; any history of angina, myocardial infarction, or coronary artery bypass graft surgery |
| Diabetes mellitus | Per chart review; insulin versus non-insulin dependent |
| End stage kidney disease | Per chart review; on hemodialysis or peritoneal dialysis |
| Hypertension | Per chart review |
| Malignancy | Per chart review; active malignancy (other than non-melanoma skin cancer) treated in the past year. Defined as cancer of the lung, breast, colorectal, prostate, gastric, pancreatic, melanoma, ovarian, brain, or other |
| Smoking | Per chart review; does not include vaping or smoking of non-tobacco products. Non-smoker, former smoker, current smoker |
| **Longitudinal Treatments^a^** |  |
| Mechanical ventilation | Invasive mechanical ventilation |
| Kidney replacement therapy | Continuous KRT, intermittent hemodialysis, peritoneal dialysis, other |
| PaO_2_^b^ | Lowest PaO_2_ available during each 24 hour day (midnight to midnight) |
| FiO_2_^b^ | FiO_2_ corresponding to the lowest PaO_2_ |
| PEEP^b^ | Highest PEEP available during each 24 hour day (midnight to midnight) |
| Vasopressors | Maximum number of vasopressors required each day |

Abbreviations: AKI, acute kidney injury; KRT, kidney replacement therapy, eGFR, estimated glomerular

filtration rate; FiO2, fraction of inspired oxygen; PaO2, partial pressure of oxygen; PEEP, positive end-expiratory pressure.

^a^Longitudinal treatments and outcomes were recorded daily for the first 14 days following admission to

the ICU. If multiple values were present, the lowest PaO2 available, along with the corresponding FiO2 at

the time, was recorded, while the highest PEEP on each day was recorded. If the patient had an

outcome, the date of the outcome was recorded.

^b^Only applies to patients on mechanical ventilation with an arterial blood gas available.

**S3 Table. Multivariable-Adjusted Risk Model for Death at 28 Days**

| **Characteristic** | **Odds Ratio (95% CI)** |
| --- | --- |
| Hispanic (vs. Non-Hispanic White) | 1.44 (1.12-1.84) |
| Age, per one year increase | 1.04 (1.03-1.05) |
| Sex, Male | 1.75 (1.36-2.25) |
| Body mass index ≥30 kg/m^2^ | 1.00 (0.79-1.28) |
| Current smoker | 1.08 (0.83-1.40) |
| Hypertension | 1.17 (0.89-1.53) |
| Diabetes | 1.19 (0.93-1.51) |
| Chronic obstructive pulmonary disease | 1.19 (0.77-1.83) |
| Coronary artery disease | 1.30 (0.92-1.83) |
| Congestive heart failure | 1.03 (0.69-1.53) |
| Chronic kidney disease | 1.18 (0.82-1.69) |
| Symptom duration prior to ICU admission, per one day increase | 0.99 (0.97-1.01) |
| D-dimer, <1000 ng/mL | 0.47 (0.36-0.62) |
| PaO_2_:FiO_2_, mm Hg, not mechanically ventilated | 0.47 (0.36-0.60) |
| Lymphocyte count, <1000/µL | 1.26 (0.99-1.62) |
| Renal component of SOFA score |  |
| 0 | 1 (Reference) |
| 1 | 1.92 (1.20-3.07) |
| 2 | 2.38 (1.13-5.00) |
| 3 | 2.00 (1.36-2.93) |
| 4 | 1.69 (1.26-2.27) |
| Hospital size (no. pre-COVID ICU beds), 50-99 | 1.12 (0.85-1.48) |
| Angiotensin-converting enzyme inhibitor | 0.90 (0.66-1.24) |
| Angiotensin receptor blocker | 1.07 (0.76-1.50) |
| Non-steroidal anti-inflammatory drug | 0.84 (0.57-1.24) |
| Aspirin | 1.40 (1.05-1.88) |
| Vitamin D | 1.22 (0.86-1.73) |
| Abbreviations: ICU, intensive care unit; IQR, interquartile range; PaO_2_:FIO_2_, ratio of PaO_2_ over the fraction of inspired oxygen (assessed only in patients receiving invasive mechanical ventilation); SOFA, sequential organ failure assessment score. | |
