# Supplementary material for: Hispanic ethnicity and mortality among critically ill patients with COVID-19
Source: PLoS One. 2022 May 18;17(5):e0268022. doi: 10.1371/journal.pone.0268022 (PMC9116663; doi:10.1371/journal.pone.0268022)
Supplement: S2 Table — (DOCX) [file pone.0268022.s004.docx]

**S2 Table. Definitions of Baseline Characteristics, Comorbidities, Treatments, and Outcomes**

| **Baseline Characteristics** |  |
| --- | --- |
| Baseline serum creatinine | Lowest value (mg/dl) within 365 to 7 days prior to hospital admission. If not available, serum creatinine on hospital admission |
| Home medications | Medications that the patient was taking at home within 1 week prior to admission. Does not include those started at an outside hospital if the patient was transferred. |
| **Coexisting Conditions** |  |
| Asthma | Per chart review |
| Chronic kidney disease | Baseline eGFR< 60 on at least two consecutive values at least 12 weeks apart prior  to hospital admission. If not available, defined as per chart review. |
| Chronic liver disease | Cirrhosis, alcohol-related liver disease, nonalcoholic fatty liver disease, autoimmune hepatitis, hepatitis B or hepatitis C, primary biliary cirrhosis, or other |
| Chronic obstructive pulmonary disease | Per chart review |
| Congestive heart failure | Per chart review; heart failure with preserved versus reduced ejection fraction |
| Coronary artery disease | Per chart review; any history of angina, myocardial infarction, or coronary artery bypass graft surgery |
| Diabetes mellitus | Per chart review; insulin versus non-insulin dependent |
| End stage kidney disease | Per chart review; on hemodialysis or peritoneal dialysis |
| Hypertension | Per chart review |
| Malignancy | Per chart review; active malignancy (other than non-melanoma skin cancer) treated in the past year. Defined as cancer of the lung, breast, colorectal, prostate, gastric, pancreatic, melanoma, ovarian, brain, or other |
| Smoking | Per chart review; does not include vaping or smoking of non-tobacco products. Non-smoker, former smoker, current smoker |
| **Longitudinal Treatments^a^** |  |
| Mechanical ventilation | Invasive mechanical ventilation |
| Kidney replacement therapy | Continuous KRT, intermittent hemodialysis, peritoneal dialysis, other |
| PaO_2_^b^ | Lowest PaO_2_ available during each 24 hour day (midnight to midnight) |
| FiO_2_^b^ | FiO_2_ corresponding to the lowest PaO_2_ |
| PEEP^b^ | Highest PEEP available during each 24 hour day (midnight to midnight) |
| Vasopressors | Maximum number of vasopressors required each day |

Abbreviations: AKI, acute kidney injury; KRT, kidney replacement therapy, eGFR, estimated glomerular

filtration rate; FiO2, fraction of inspired oxygen; PaO2, partial pressure of oxygen; PEEP, positive end-expiratory pressure.

^a^Longitudinal treatments and outcomes were recorded daily for the first 14 days following admission to

the ICU. If multiple values were present, the lowest PaO2 available, along with the corresponding FiO2 at

the time, was recorded, while the highest PEEP on each day was recorded. If the patient had an

outcome, the date of the outcome was recorded.

^b^Only applies to patients on mechanical ventilation with an arterial blood gas available.
