# Supplementary material for: Hispanic ethnicity and mortality among critically ill patients with COVID-19
Source: PLoS One. 2022 May 18;17(5):e0268022. doi: 10.1371/journal.pone.0268022 (PMC9116663; doi:10.1371/journal.pone.0268022)
Supplement: S3 Table — (DOCX) [file pone.0268022.s005.docx]

**S3 Table. Multivariable-Adjusted Risk Model for Death at 28 Days**

| **Characteristic** | **Odds Ratio (95% CI)** |
| --- | --- |
| Hispanic (vs. Non-Hispanic White) | 1.44 (1.12-1.84) |
| Age, per one year increase | 1.04 (1.03-1.05) |
| Sex, Male | 1.75 (1.36-2.25) |
| Body mass index ≥30 kg/m^2^ | 1.00 (0.79-1.28) |
| Current smoker | 1.08 (0.83-1.40) |
| Hypertension | 1.17 (0.89-1.53) |
| Diabetes | 1.19 (0.93-1.51) |
| Chronic obstructive pulmonary disease | 1.19 (0.77-1.83) |
| Coronary artery disease | 1.30 (0.92-1.83) |
| Congestive heart failure | 1.03 (0.69-1.53) |
| Chronic kidney disease | 1.18 (0.82-1.69) |
| Symptom duration prior to ICU admission, per one day increase | 0.99 (0.97-1.01) |
| D-dimer, <1000 ng/mL | 0.47 (0.36-0.62) |
| PaO_2_:FiO_2_, mm Hg, not mechanically ventilated | 0.47 (0.36-0.60) |
| Lymphocyte count, <1000/µL | 1.26 (0.99-1.62) |
| Renal component of SOFA score |  |
| 0 | 1 (Reference) |
| 1 | 1.92 (1.20-3.07) |
| 2 | 2.38 (1.13-5.00) |
| 3 | 2.00 (1.36-2.93) |
| 4 | 1.69 (1.26-2.27) |
| Hospital size (no. pre-COVID ICU beds), 50-99 | 1.12 (0.85-1.48) |
| Angiotensin-converting enzyme inhibitor | 0.90 (0.66-1.24) |
| Angiotensin receptor blocker | 1.07 (0.76-1.50) |
| Non-steroidal anti-inflammatory drug | 0.84 (0.57-1.24) |
| Aspirin | 1.40 (1.05-1.88) |
| Vitamin D | 1.22 (0.86-1.73) |
| Abbreviations: ICU, intensive care unit; IQR, interquartile range; PaO_2_:FIO_2_, ratio of PaO_2_ over the fraction of inspired oxygen (assessed only in patients receiving invasive mechanical ventilation); SOFA, sequential organ failure assessment score. | |
